# Heterogeneous Nucleation of Supersaturated Water Vapor onto Sub-10 nm Nanoplastic Particles

**DOI:** 10.1021/acs.est.2c07643

**Published:** 2023-01-19

**Authors:** Peter J. Wlasits, Ruth Konrat, Paul M. Winkler

**Affiliations:** † Faculty of Physics, University of Vienna, Vienna 1090, Austria; ‡ Vienna Doctoral School in Physics, University of Vienna, Vienna 1090, Austria

**Keywords:** heterogeneous nucleation, nanoplastic, nucleation probability, water vapor/aerosol, saturation ratio, polyethylene terephthalate (PET), Size Analyzing Nuclei Counter

## Abstract

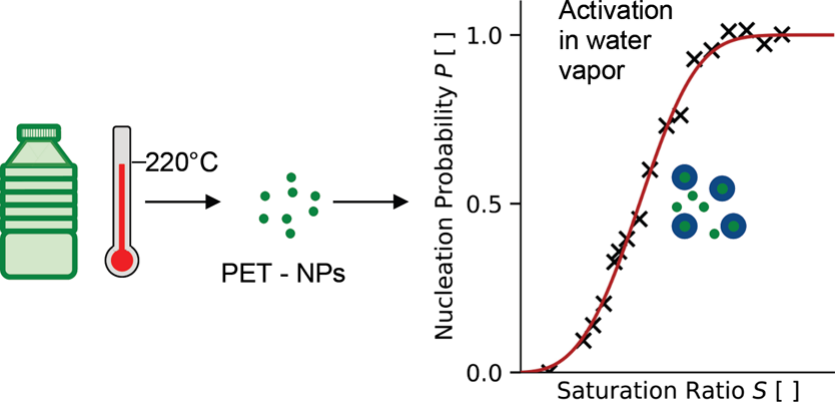

Nanoplastic pollution by atmospheric transport processes is a recently discovered environmental problem on a global scale that is attributed to the dispersion of aerosolized nanoplastics. However, knowledge about the basic physicochemical properties of aerosol nanoplastic particles is scarce. Here, we present experiments on the heterogeneous nucleation of supersaturated water vapor onto sub-10 nm polyethylene terephthalate (PET) seeds. We determined onset saturation ratios for the activation of PET seeds in comparison to the well-documented reference system of silver particles, resulting in lower onset saturation ratios of the PET seeds compared to silver seeds. By using different PET bulk materials for the generation of nanoparticles, we report a strong material dependence of the onset saturation ratios, pointing to a strong effect of additives from commodity plastics in heterogeneous nucleation. Moreover, our results show a strong dependence on nucleation temperature that might be of immediate atmospheric relevance. Our work can be considered as an initial step in airborne nanoplastic detection by condensation techniques, and we anticipate our study to serve as a basis for further research that will eventually allow assessing the impact of nanoplastic dispersion on atmospheric processes.

## Abstract 2

Process-level studies are needed to understand environmental dispersion of nanoplastics. Here we address heterogeneous nucleation of water vapor on nanoplastic aerosols relevant for cloud processing and airborne detection of nanoplastics.

## Introduction

Since the 1970s, atmospheric aerosols are known to be a major anthropogenic driver of climate change.^1^ Aerosol particles in the atmosphere interact directly with solar radiation and indirectly by serving as cloud condensation nuclei (CCN) as well as ice nuclei (IN) upon which cloud droplets and ice crystals form.^2−4^ Especially the indirect radiative effect depends strongly on the abundance of CCN, which is influenced by new particle formation.^5,6^

In recent years, micro- and nanoplastics have been identified to be novel pollutants of anthropogenic origin.^7^ In general, microplastics are particles with diameters between 1 μm and 5 mm, whereas nanoplastics refer to particles with diameters smaller than 1 μm.^8,9^ The dispersion of nanoplastic particles across various ecosystems^10^ clearly indicates that pollution with micro- and nanoplastics is an environmental problem on a global scale. A growing body of literature has reported plastic particles in sparsely inhabited regions, spanning from a remote high-altitude site in Austria^11^ to the Arctic,^7,12^ thereby emphasizing the importance of atmospheric transport processes for such plastic particles.^13^

Nanoplastics in particular are believed to constitute a significant proportion of the plastics debris budget.^7^ Commonly, nanoplastics are perceived as submicron particles originating from the fragmentation and degradation of microplastics.^14,15^ However, under natural conditions nanoplastic breakdown is a long-term process of several months to years,^16^ that is eventually inhibited by the increasing dominance of London–van der Waals forces with decreasing particle size. Recent research has also uncovered thermal stress as primary source of nanoplastic particles.^17,18^ Contrary to fragmentation and degradation processes, abundant nanoparticles with diameters below 100 nm were found to be directly released within seconds.^17^ Evidently, current methods are blind to these small nanoplastic particles. In this size range, however, indirect radiative effects via cloud formation as well as health effects play a potentially crucial role.

Moreover, nanoplastics are effective ice-nucleating particles and suspected to interact with other natural and anthropogenic contaminants in the atmosphere.^19,20^ In particular, the high surface-to-volume ratio of nanoplastics affects their reactivity and the way other pollutants can be absorbed.^21^ Consequently, atmospheric nanoplastic particles might influence cloud formation^20,22^ as well as scatter and absorb radiation thereby affecting the Earth’s effective radiative forcing.^7^

Despite perpetually growing research interest, atmospheric transport processes of plastic particles are still poorly understood,^23^ because the detection and characterization of airborne nanoplastics are thus far not possible.^21^ The development of new sampling and characterization techniques is needed to deliver first insights into crucial properties of nanoplastic aerosols, like particle sizes and abundance. Recent research has shown that aerosolized nanoplastics can be reproducibly generated from the controlled evaporation–condensation of macroplastics,^24^ thereby enabling process-level studies of heterogeneous nucleation onto nanoplastic seeds.

Heterogeneous nucleation processes are phase transitions of metastable vapors that form a new liquid phase on the surface of airborne particles.^25^ Heterogeneous nucleation can occur on ions and soluble or insoluble particles and is energetically easier than homogeneous nucleation, which is the formation of new particles from the vapor phase in the absence of any seeds.^25,26^ The surface of preexisting particles reduces the energy barrier necessary to allow for the phase transition.^25^ Since fundamental atmospheric phenomena are driven by these processes, heterogeneous nucleation is of high importance for aerosol research.^27^ Cloud droplets in the lower atmosphere, for example, form due to heterogeneous nucleation of water vapor onto aerosol particles.^3,28^ Moreover, heterogeneous nucleation and subsequent condensational growth enable the detection of nanoscopic particles in condensation particle counters (CPCs).^29^ Due to the complexity of interactions between nucleating molecules and the underlying surfaces, quantification of heterogeneous nucleation is oftentimes difficult.^30,31^

Here, we present the first results of a set of experiments on the heterogeneous nucleation of water vapor onto sub-10 nm seed particles originating from polyethylene terephthalate (PET).

## Materials and Methods

The seed particles used in the experiments were generated from polyethylene terephthalate (PET, (C_10_H_8_O_4_)_n_), a common thermoplastic material. Commodity and higher-purity versions of PET were used. Commodity PET was harvested from a water bottle and chipped into platelets using a hole puncher.^24^ Unlike the commodity PET in use, higher-purity PET was plastic of known composition and additive content. More detailed information about the thermoplastics used can be found in Wlasits et al.^24^ Silver (Ag) seeds were used as a reference material. A table containing the CAS registry numbers as well as purity grades of the bulk materials used for the experiments is presented in the Supporting Information (SI, Table S1)

**Figure 1:**
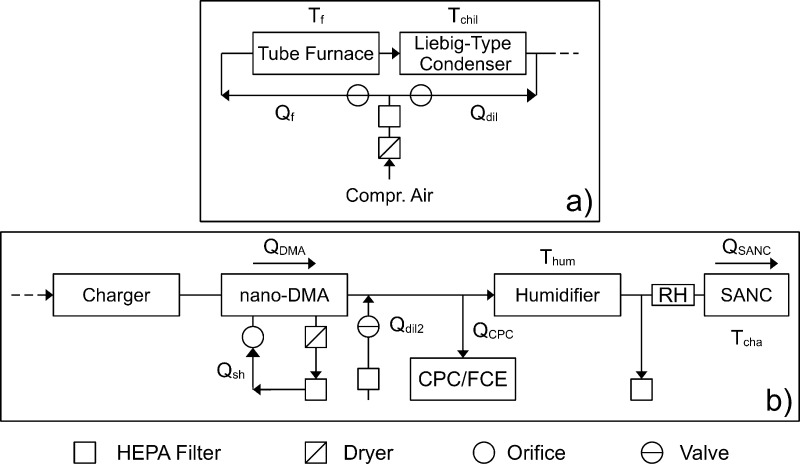
The figure presents a schematic of the experimental setup. Panel (a) shows the setup used for particle generation, which is based on material evaporation in a tube furnace. Panel (b) presents a schematic of the experimental setup used for size selection of the generated particles, humidification of the aerosol, as well as particle detection. A Vienna-type nano-DMA was used to select particles based on their electrical mobility equivalent diameter. Particle number concentrations were determined upstream of the humidifier using a CPC or an FCE. In order to determine the relative humidity of the aerosol a sensor was placed upstream of the SANC. Flow rates are denoted by *Q*. Temperatures are denoted by *T*. Apart from the PID-controlled temperature of the tube furnace *T*_*f*_, temperatures were maintained with thermostats.

Figure 1 shows a schematic of the experimental setup. Particle-free, compressed, and dried air was used as carrier gas in all experiments. A quartz glass crucible was filled with bulk material and placed centrally inside the working tube of the tube furnace (Nabertherm R50/250/13, Nabertherm GmbH, Lilienthal, Germany). By heating the furnace, the material is evaporized.^32^ A constant flow of carrier gas transports the material vapor into cooler regions of the tube furnace. Rapid cooling using a Liebig-type condenser leads to particle formation based on homogeneous nucleation.^32^ The temperature of the water flushing the condenser was maintained using a thermostat (Lauda, Model RMS 6, Dr. R. Wobser GmbH & Co KG, Koenigshofen, Germany). Depending on the experiment, the generated aerosol was diluted with compressed and dried air downstream of the condenser (s. Figure 1a). Since the generation method is prone to contaminations,^24^ the glass tubes in use were inspected on a regular basis. In case of Ag particles, a smaller makeshift tube furnace, composed of a PID controller (ESM-4430, Emko Elektronik A.S., Bursa, Turkey) and a 1200 W-DC power supply (QPX1200SP, Aim and Thurlby Thandar Instruments Ltd., Cambridgeshire, United Kingdom) was used for aerosol generation. Consequently, any contamination of the Ag particles by PET can be excluded.

Downstream of the particle generator a soft X-ray charger (Advanced Aerosol Neutralizer 3088, TSI Inc., Shoreview, USA) was used to establish a steady-state charge distribution.^24,33,34^ Subsequent to charging, the aerosol was fed into a custom-made Vienna-type differential mobility analyzer (nano-DMA^26,35^) thereby enabling particle selection based on their electrical mobility. The geometrical parameters of the nano-DMA in use are summarized in the SI (s. Table S2). Positive voltages were applied to the nano-DMA and, as a consequence, negatively charged particles were sampled.

A closed-loop sheath air cycle was used for DMA operation. The sheath air cycle was composed of a pump, a silica gel dryer, and a HEPA filter. The flow rate of dried and particle-free sheath air was maintained using a critical orifice (s. Figure 1b). The relative humidity (RH) of the sheath air cycle as well as the RH of the aerosol exiting the nano-DMA was closely monitored to ensure optimal experimental conditions (RH < 10 %). All experiments were conducted using a sheath air flow rate (*Q*_*sh*_) of 29.7 L·min^-1^ or 30.7 L·min^-1^ depending on the mobility diameter and the particle number concentrations (PNCs) of the seed particles. PNCs were determined downstream of the DMA using a CPC (TSI 3776) in order to monitor the stability of the particle generation unit. A Faraday cup electrometer (FCE, TSI 3068B) was occasionally used for sub-5 nm particles or elevated PNCs (> 3 × 10^5^ cm^-3^).

The monodisperse aerosol was then fed into a diffusion-type humidifier. The humidifier used during the experiments was composed of a double-walled glass tube with a length of 1.01 m and an inner diameter of 0.04 m. The inner walls were lined with two layers of blotting paper that were regularly soaked with HPLC-grade water. The water vapor content of the exiting aerosol was controlled by adjusting the humidifier temperature (*T*_*hum*_). The temperature of the humidifier was controlled using a thermostat (Lauda, Model RKS 20-D, Dr. R. Wobser GmbH & Co KG, Koenigshofen, Germany). Comparatively high aerosol flow rates were required to transport a sufficient number of particles through the setup resulting in undersaturated aerosol exiting the humidifier. Consequently, the relative humidity of the aerosol was monitored downstream of the humidifier using an RH sensor (SHT75, Sensirion AG, Staefa, Switzerland, s. Table S3). Since accurate measurements of the RH of the aerosol exiting the humidifier, the temperature of the humidifier *T*_*hum*_ and the temperature of the chamber *T*_*cha*_ are needed for the calculation of the saturation ratio after expansion, the aerosol–vapor mixture is given time to thermally equilibrate with the expansion chamber prior to any measurements.^36^

Subsequent to preconditioning and humidification, the aerosol was fed into the Size Analyzing Nuclei Counter (SANC). The SANC is a pressure-defined expansion chamber that is used to study heterogeneous nucleation and particle growth in supersaturated vapors.^30,36,37^ The underlying measuring principle of the SANC is constant-angle Mie scattering (CAMS^37^). Growing particles in the expansion chamber are illuminated by a beam of monochromatic and parallel light. The transmitted light flux as well as the light flux scattered under a constant angle are measured. The experimental data are compared with the corresponding light fluxes calculated according to Mie theory.^36^ Heterogeneous nucleation and subsequent condensational particle growth is obtained by adiabatic expansion of the aerosol–vapor mixture held at the temperature of the measurement chamber. Based on precise knowledge of the initial conditions (chamber temperature *T*_cha_, partial vapor pressure and total gas pressure), Poisson’s law is used to calculate the temperature and saturation ratio after the expansion.^25,38^ The saturation ratio *S* is defined as the ratio of the partial vapor pressure to the equilibrium vapor pressure at a certain temperature. The temperature *T*_0_ in the chamber right after the expansion is referred to as the nucleation temperature. *T*_cha_ is controlled using a thermostat (Lauda, Variocool VC1200, Dr. R. Wobser GmbH & Co KG, Koenigshofen, Germany). A more detailed description of the SANC can be found in Winkler et al.^36^

Vapor supersaturation is increased from zero particle activation to full particle activation. The nucleation probability (*P*_nuc_) is then given by
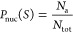
1*N*_a_ denotes the number of activated seeds, and *N*_tot_ refers to the total number of seed particles. The onset saturation ratio at the nucleation temperature *S*_0_(*T*_0_) then corresponds to a nucleation probability of 50%. The experimentally determined saturation ratio is cross-checked by comparison of experimental and theoretical droplet growth curves, which are highly sensitive to the saturation ratio and provide a robust measure of uncertainty (s. SI, Figure S1). The capability to precisely determine the saturation ratio makes SANC a unique instrument for quantitative studies of heterogeneous nucleation.

## Results and Discussion

The experimental approach allows for the determination of nucleation probabilities for seed particles of known electrical mobility diameter. Simultaneously, the saturation ratios of the water vapor as well as the nucleation temperatures are recorded. The onset saturation ratios are then determined by fitting the experimental nucleation probabilities. Consequently, the main results of the presented study consist of onset saturation ratios *S*_0_ for size-selected seed particles at a certain nucleation temperature *T*_0_. For optimal experimental conditions, the total PNCs of activated particles were kept between 10,000 cm^-3^ and approximately 30,000 cm^-3^ inside of the measurement chamber. In order to maintain decent PNCs as well as to minimize deviations from the desired nucleation temperature, every measurement run was based on individual experimental settings, e.g., flow rate of air passing the tube furnace and setpoint temperature of the tube furnace. The settings corresponding to the presented data are summarized in the SI (Tables S4 and S5).

Unlike a number of previous studies, that used a syringe pump for vapor generation, e.g., Kupc et al.^31^ and Schobesberger et al.,^39^ our experimental approach relied on the direct measurement of the RH of the preconditioned aerosol. Consequently, the setup was tested by reproducing the results of previous measurements. Measurements using negatively charged Ag seeds were compared to the results published by Kupc et al.^31^ and satisfactory agreement between the two studies was found (s. SI, Figure S2).

### Nucleation Probabilities of Higher-Purity PET Seeds

**Figure 2:**
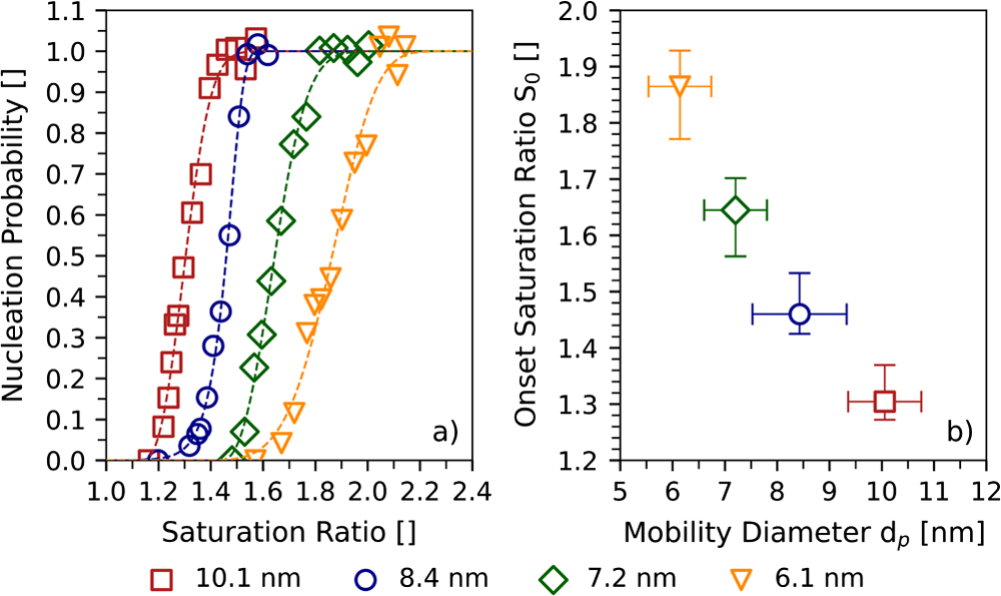
Nucleation probabilities and onset saturation ratios from higher-purity PET. Panel (a) shows the nucleation probabilities versus the determined saturation ratios of water vapor for PET^P^ particles with mobility diameters between 6.1 and 10.1 nm. Panel (b) presents the corresponding onset saturation ratios *S*_0_. The onset saturation ratios were determined at a nucleation temperature of 11 ± 1 °C. The onset saturation ratios correspond to a nucleation probability of 50% and were determined by fitting the activation curves in Figure 2a.

Figure 2 shows the nucleation probabilities measured using particles originating from higher-purity PET (PET^P^). The mobility diameters of the seeds ranged from 6.1 to 10.1 nm and the nucleation temperature was maintained at 11 ± 1 °C. Figure 2a presents the measured nucleation probability as a function of the saturation ratio of the water vapor. The activation curves confirm the trend for decreasing particle sizes as predicted by the classical nucleation theory: The activation of seed particles with decreasing mobility diameters requires gradually increasing saturation ratios.

Figure 2b presents the onset saturation ratio *S*_0_ as a function of the electrical mobility diameter *d*_p_. As expected, the onset saturation ratios increase with decreasing seed particle diameters. Despite being measured under controlled laboratory conditions, the results presented in Figure 2 are highly relevant for research on atmospheric nanoplastic particles. Particles derived from macroscopic PET samples under thermal stress can be activated in water vapor. Although the measured onset saturation ratios are larger than the saturation ratios expected in the atmosphere, it must be noted that these onset saturation ratios mainly depend on particle size. Since the onset saturation ratios decrease with increasing particle size, a potential impact of nanoplastics on cloud formation should be considered. Extrapolation of our experimental data toward larger particle sizes using a Kelvin-type exponential function suggests that particles of diameters as small as 20 nm can be activated to growth under atmospherically relevant supersaturations. It is therefore plausible that nanoplastic particles in the range of CCN sizes under otherwise identical conditions can be activated to cloud droplets. Reliable detection methods are needed to accurately assess the concentrations of these particles in the atmosphere.

Particular attention was given to the determination of the measurement uncertainties of the data in Figure 2b. Measurement uncertainties are mainly influenced by the accuracy of the RH sensor in use as well as by condensational growth analysis.^40^ Both sources of uncertainty were addressed for the calculation of the uncertainty bars. Thus, the uncertainty bars were determined such that they reflect the largest uncertainties arising either from the RH sensor or from the growth model. Numerical values for the uncertainties are presented in the SI (s. Table S4).

### Comparison to Commodity PET and Ag Seeds

**Figure 3:**
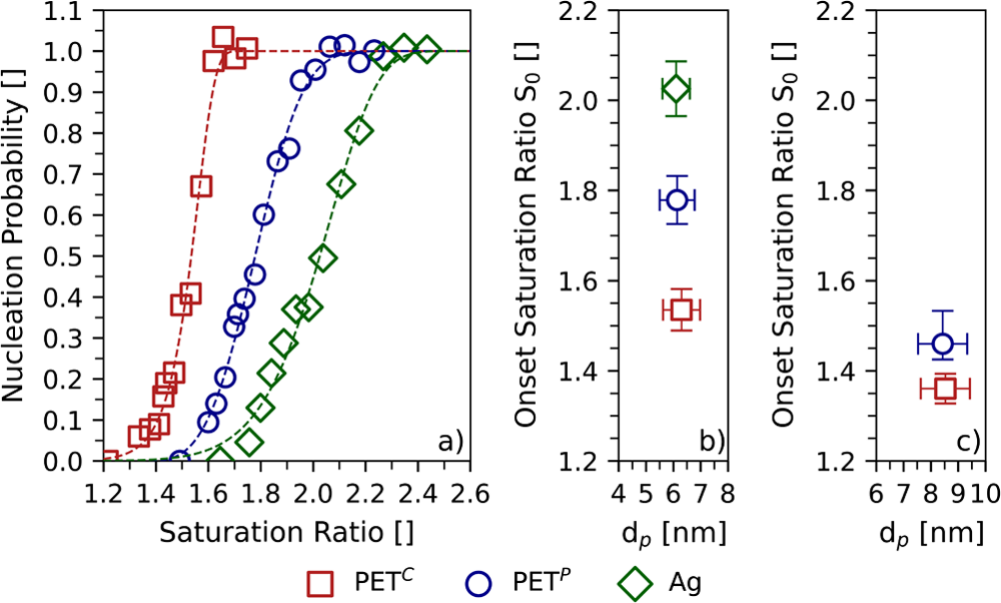
Nucleation probabilities as well as the determined onset saturation ratios for higher-purity PET, commodity PET, and silver seeds. Panel (a) shows the nucleation probabilities versus the determined saturation ratio of water vapor for seed particles with mobility diameters of approximately 6 nm at a nucleation temperature of roughly 7.5 °C. Panel (b) presents the onset saturation ratios *S*_0_ as obtained from fit curves in Figure 3a. Panel (c) presents the onset saturation ratios *S*_0_ for PET seed particles with mobility diameters of approximately 8.5 nm at a nucleation temperature of roughly 11.5 °C.

In the next step, the activation of PET^P^ seeds was compared with measurements using seeds generated from platelets of commodity PET (PET^C^) and silver wool. Figure 3a presents the corresponding activation curves. The activation curves were measured for particles with mobility diameters of approximately 6 nm at a nucleation temperature of 7.5 ± 0.3 °C. Substantial differences in the activation of PET^P^, PET^C^, and Ag seeds are evident. Figure 3b shows these differences in particle activation in terms of the onset saturation ratios. Interestingly, seed particles generated from PET^C^ are activated at significantly lower saturation ratios than PET^P^ seeds. Ag particles yielded the highest onset saturation ratio.

Based on these results the difference in the activation of PET^P^ and PET^C^ seeds was investigated in further detail by performing measurements with larger seeds and at a higher nucleation temperature as illustrated in Figure 3c. The experiments were conducted with particles having mobility diameters of around 8.5 nm while maintaining a nucleation temperature of approximately 11.5 °C. The most conspicuous result to emerge from these measurements with changed settings is that the difference in the onset saturation ratio of PET^P^ and PET^C^ seeds is significantly smaller for larger particles at a higher nucleation temperature. Taking into account the center values, the difference of the onset saturation ratio *S*_0_ between the two plastic seeds yields 0.25 for 6 nm particles at the lower nucleation temperature. For 8.5 nm particles at a nucleation temperature of 11.5 °C the difference of *S*_0_ diminishes to 0.1.

The observed differences in particle activation can be compared to the results published by Wlasits et al.:^24,35^ The authors report that the 50% cutoff diameters measured for PET^P^ and PET^C^ seeds using a butanol-based CPC are almost equal and significantly smaller than the 50% cutoff diameters measured using negatively charged NaCl seeds.^24^ PET seeds appeared to be chemically more similar to n-butanol than to polar solvents, like water.^24^ As a consequence, it can be expected that, compared to NaCl, PET seeds are activated at higher onset saturation ratios in polar vapors.^35^

Our results confirm these findings: Compared to onset saturation ratios measured using neutralized NaCl seeds,^31^ we report significantly higher onset saturation ratios for negatively charged Ag, PET^P^, and PET^C^ seeds using water as condensable vapor. Ag seeds were activated at significantly higher onset saturation ratio than PET^P^ and PET^C^ seeds. Although oxidation during the generation of Ag seeds can be expected, PET^P^ and PET^C^ seeds originate from polyesters, thereby providing many docking stations to molecules of the working fluid.^41−43^ These docking stations can be assumed to enhance heterogeneous nucleation processes.^41^

Unlike Wlasits et al.,^24^ we report considerable disparity in the activation of PET^P^ and PET^C^ seeds. The results presented in Figure 3 indicate that these differences are caused by an effect of the nucleation temperature and point toward differences in the chemical composition of the seeds under investigation. An effect based on the setpoint temperature of the tube furnace during particle generation cannot be excluded (s. SI, Table S5). Thus, comparisons between our study and the study by Wlasits et al.^24^ must be treated with caution.

### Effect of the Nucleation Temperature

**Figure 4:**
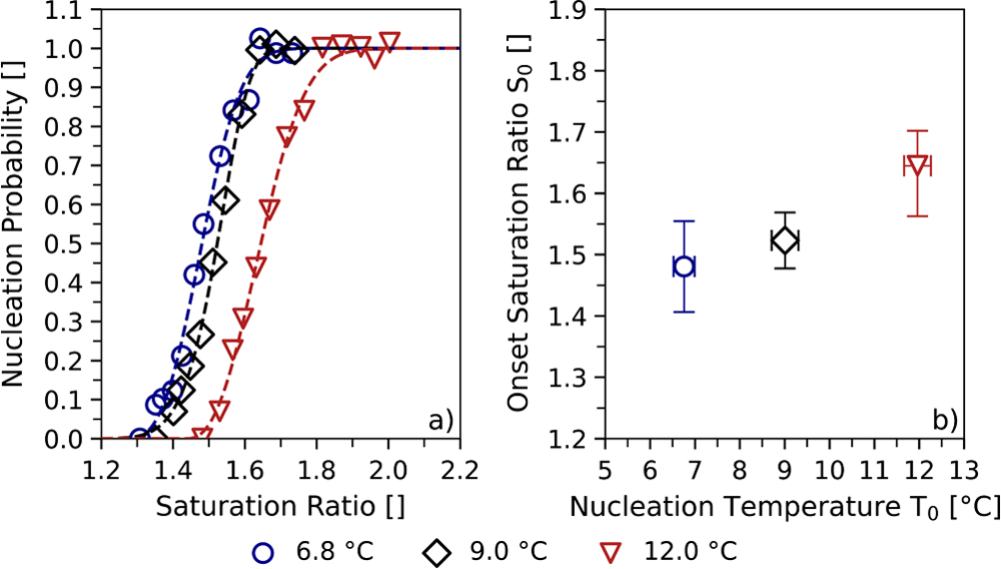
Nucleation probabilities as well as the determined onset saturation ratios of PET^P^ seeds at three different nucleation temperatures ranging from 6.8 to 12.0 °C. The seeds used for the experiments had mobility diameters of 7.2 nm. Panel (a) presents the measured activation curves. The onset saturation ratio *S*_0_ as a function of the nucleation temperature is shown in panel (b).

Taking into account the results presented in Figure 3, the next consecutive step was composed of measurements at different nucleation temperatures. Figure 4a clearly shows that an increase of the nucleation temperature at constant seed particle diameter yields a significant shift of the activation curves toward higher saturation ratios. While the curves corresponding to a nucleation temperature of 6.8 and 9.0 °C only show a small difference, the curve relating to a nucleation temperature of 12.0 °C is clearly separated. Based on the measurements, lower nucleation temperatures appear to favor particle activation, thereby decreasing the necessary onset saturation ratios (s. Figure 4b).

**Figure 5:**
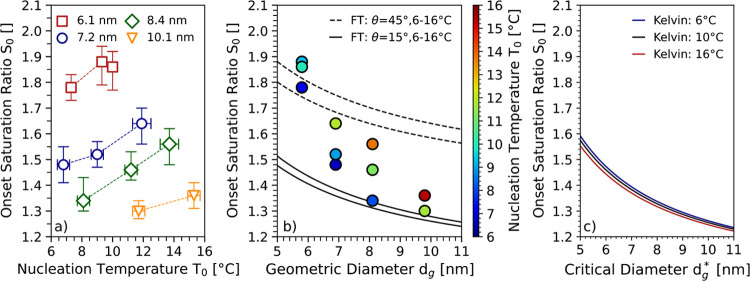
Onset saturation ratios at different nucleation temperatures using PET^P^ seeds with diameters ranging from 6.1 to 10.1 nm. Panel (a) presents the onset saturation ratio versus the nucleation temperature. The markers correspond to differently sized seed particles and the dashed lines are solely a guide to the eye. Panel (b) presents the onset saturation ratio as a function of the geometric diameter. The colors of the markers indicate the nucleation temperature. The solid and dashed lines correspond to theoretical onset saturation ratios calculated from Fletcher theory (FT) for different contact angles θ (15° and 45°) at a temperature range slightly exceeding the experimental nucleation temperatures. For the sake of readability, uncertainty bars were removed in panel (b). Panel (c) shows the onset saturation ratios as a function of the critical geometric diameter *d*_g_^*^ as predicted by the Kelvin equation.

To verify the aforementioned observation, a set of measurements was conducted that included a wider range of seed particle diameters. The results are summarized in Figure 5. The figure presents data based on measurements using PET^P^. The same graph for PET^C^ particles can be found in the SI (s. Figure S3).

Figure 5a presents the onset saturation ratios of seed particles with mobility diameters ranging from 6.1 to 10.1 nm as a function of the nucleation temperature. Similar to the temperature trend shown in Figure 4a, increasing onset saturation ratios for increasing nucleation temperatures were also found for particles with larger diameters (8.4 and 10.1 nm).

Experimental results are compared with the predictions from Fletcher theory^44^ (FT, s. SI) in Figure 5b. The theory introduces a contact angle θ to describe interactions between the condensing vapor and the surface of perfectly spherical seeds.^25,44^ Hence, Fletcher theory assists to assess whether wetting phenomena are causing the differences in the onset saturation ratios for different seed particle diameters and nucleation temperatures. The solid and dashed lines in Figure 5b correspond to theoretical predictions. It must be noted that the mobility equivalent diameter based on size selection using a DMA is assumed to be approximately 0.3 nm larger than the geometric diameters used in FT.^25,45^ As a consequence, the mobility diameters of the seed particles have been corrected using the aforementioned approximation.

A contact angle θ of roughly 15° has been reported by Winkler et al.^46^ for hydrophobic Ag seeds with particle diameters of approximately 6.5 nm. The contact angle was therefore successively increased in order to fit the data. Comparison to theoretical predictions revealed significant differences between experimental data and theory. The theoretical curves based on two different contact angles (15° and 45°) predict distinctively smaller increases in the onset saturation ratios toward smaller mobility diameters as measured during this study. Consequently, our results demonstrate that neither taking into account the contact angle from FT nor the considered temperature range is sufficient for an accurate explanation of the experimental data. Figure 5b suggests that the contact angle θ depends on temperature as discussed by McGraw et al.^47^ and Song and Fan.^48^ This hypothesis could be investigated by direct determination of the contact angle as outlined by Winkler et al.^46^ Hence, future research should include nanoplastic seeds that are size-selected using a high-resolution DMA^49,50^ prior to activation.

Remarkably, the observed temperature trend indicates that nanoplastic particles may become candidates for CCNs at cold conditions despite their usually hydrophobic behavior. To assess the significance of the observed temperature-dependent onset saturation ratios the results are compared to predictions based on Kelvin equation^51^ in Figure 5c. As expected, Kelvin equation underestimates the observed temperature-induced increase of the onset saturation ratios to a considerable degree and also predicts the opposite temperature trend. When comparing the results to prediction of FT and Kelvin equation, it needs to be kept in mind that both theoretical approaches rely on spherical particles. Accordingly, for the presented comparisons spherical seed particles were assumed.

**Figure 6:**
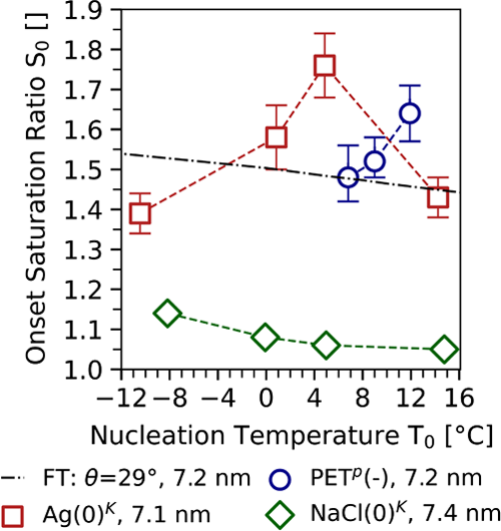
Onset saturation ratios of negatively charged PET^P^ as well as neutralized silver and sodium chloride seeds at different nucleation temperatures. Data based on Ag and NaCl seeds were taken from Kupc et al.^31^ and are marked with superscript K. The dash-dotted line corresponds to theoretical onset saturation ratios following Fletcher theory. For the sake of readability, the uncertainty bars linked to the nucleation temperatures have been removed. All numerical values including all measurement uncertainties can be found in the SI (s. Table S4) and in the Supplement of Kupc et al.^31^

In the final step of analysis, experimentally retrieved data have been compared to previous studies. The study by Kupc et al.^31^ is of special interest since the authors also used water as condensable vapor. Figure 6 combines the experimental data for PET^P^ seeds presented in this study with experimental data for neutralized silver and sodium chloride seeds as presented in Kupc et al.^31^ Comparison to experimental data from Kupc et al.^31^ is possible since charge effects usually only play a role at seed diameters smaller than 5 nm.^26^

The figure shows the experimentally determined onset saturation ratio as a function of the nucleation temperature for seed particles with an electrical mobility diameter of approximately 7.2 nm. Classical nucleation theory predicts that increasing temperatures are connected to decreasing onset saturation ratios *S*_0_.^25,47^ Remarkably the figure shows, that the temperature trend of the onset saturation ratios depends on the type of seed particle used in the experiments. Data recorded using soluble sodium chloride seeds follow the temperature trend predicted by theory. It should be noted that Köhler theory^52^ for soluble seeds as well as FT for insoluble seeds and Kelvin equation predict decreasing onset saturation ratios for increasing nucleation temperatures.^31^

On the contrary, neutralized Ag seeds and negatively charged PET^P^ seeds exhibit the opposite trend, i.e., an increase of *S*_0_ with increasing nucleation temperatures. Additionally, Kupc et al.^31^ report a maximum of *S*_0_ at nucleation temperature of about 4 °C for Ag seeds. However, our data using PET^P^ seeds show a steady increase of *S*_0_ at nucleation temperatures ranging from 6.8 to 11.9 °C.

As outlined by McGraw et al.^53^ and discussed by Kupc et al.,^31^ observations of such an unusual temperature dependence might be connected to surface properties of the seeds that the macroscopic parameters used in classical nucleation theory do not reflect.

Our results underline that a thorough understanding of heterogeneous nucleation processes requires disentanglement of the interplay between nucleation temperature, surface properties of nanoplastic particles, the chemical structure of condensing vapors, and related properties. Based on the presented results, it is undisputable that future research needs to include thorough chemical characterization of the seeds utilizing suitable methods such as mass spectroscopy. Such measurements would provide crucial insights into the role of the temperature of the tube furnace and could potentially explain the observed differences in the activation of PET^P^ and PET^C^ seeds.

As already outlined, a significant portion of the plastic debris budget is believed to be attributable to nanoplastics.^7^ Despite their hydrophobic nature, alterations of particle properties in the atmosphere have the potential to change the wettability of their surfaces.^43^ These alterations include weathering processes, oxidation reactions based on photochemical processes, and the sorption of other natural or anthropogenic compounds.^14,43^ Besides their atmospheric abundance, the formation of hydrophilic chemical groups originating from the aforementioned alterations is critical for the ability of nanoplastic particles to act as CCNs.^42,43^ Consequently, future research needs to move toward incorporating and controlling such atmospheric processes. One easily implementable approach consists of exposing the produced nanoparticles to UV radiation or ozone. Alterations of the particles’ surfaces could be mimicked by expanding the range of plastics investigated and by harvesting bulk materials from different sources. In a more general context, our study reveals that condensation techniques could be useful tools for investigating aging and weathering processes nanoplastic particles might be exposed to.

The presented experimental approach upon consecutive calibration involving a larger number of plastic-derived seeds and adaption to atmospheric conditions constitutes a globally unique platform to investigate how alterations of particle properties relate to their activation in the atmosphere. These adaptions must include arrangements of the nucleation temperatures and particle sizes such that the measured onset saturation ratios are forced into an atmospherically relevant range. Consequently, it should be emphasized that future research needs to combine all of the discussed approaches to obtain quantitative numbers of the atmospheric nanoplastic budget and to successively address potential impacts on climate and health. We therefore anticipate our study to act as a basis for further research toward the impact of nanoplastic dispersion on atmospheric processes, and to be a first step for the detection of nanoplastics in airborne state.

## Supporting Information Available

Additional details on the instruments and particle materials in use as well as tables presenting all measured data, including a brief discussion of the uncertainties and an outline of Fletcher theory (PDF)
