# Supplementary material for: Heterogeneous Nucleation of Supersaturated Water Vapor onto Sub-10 nm Nanoplastic Particles
Source: Environ Sci Technol. 2023 Jan 19;57(4):1584–91. doi: 10.1021/acs.est.2c07643 (PMC9893723; doi:10.1021/acs.est.2c07643)
Supplement: Supplementary file 1 — es2c07643_si_001.pdf [file es2c07643_si_001.pdf]

Supporting Information: Heterogeneous nucleation of supersaturated water vapor onto sub-10 nm nanoplastic particles

Peter J. Wlasits,[†,‡] Ruth Konrat,[†] and Paul M. Winkler[*,†]

†*Faculty of Physics, University of Vienna, 1090 Vienna, Austria*

‡*Vienna Doctoral School in Physics, University of Vienna, 1090 Vienna, Austria*

E-mail: paul.winkler@univie.ac.at

The Supporting Information consists of 11 pages and is composed of 5 sections presenting 3 figures and 5 tables. Section A presents additional information related to the bulk materials in use as well as the technical specifications of the DMA and the RH sensor. Supplementary results and results of calibration experiments can be found in Section B. Section C contains the complete dataset used for the figures in the manuscript. Section D presents comments on measurement uncertainties and Section E summarizes the main equations of Fletcher theory.

A. Additional Tables - Materials and Methods

Table S1: The table presents basic information about the seed particle materials used for the experiments.

Compound	Chemical Formula	CAS RN	Purity/Grade
Silver	Ag	7440-22-4	for elemental analysis
Polyehtylene Terephthalate (PET^P)	$(C_{10}H_8O_4)_n$	25038-59-9	-
Water	H_2O	7732-18-5	HPLC+

Table S2: DMA Dimensions: The table summarizes the geometrical parameters of the nano-DMA used in the experiments.

Inner Radius R_i	0.0175 m
Outer Radius R_o	0.0241 m
Length L	0.0150 m

Table S3: The table presents the specifications of the RH sensor used for the experiments based on information provided by the manufacturer[1].

Parameter	Value	Unit
Operating Range	0-100	%(RH)
Accuracy (10-90 %)	± 1.8	%(RH)
Accuracy (>90-100 %)	max. ± 4.0	%(RH)
Repeatability	± 0.1	%(RH)
Hysteresis	± 1.0	%(RH)
Response Time	8	s

B. Additional Figures - Results and Discussion

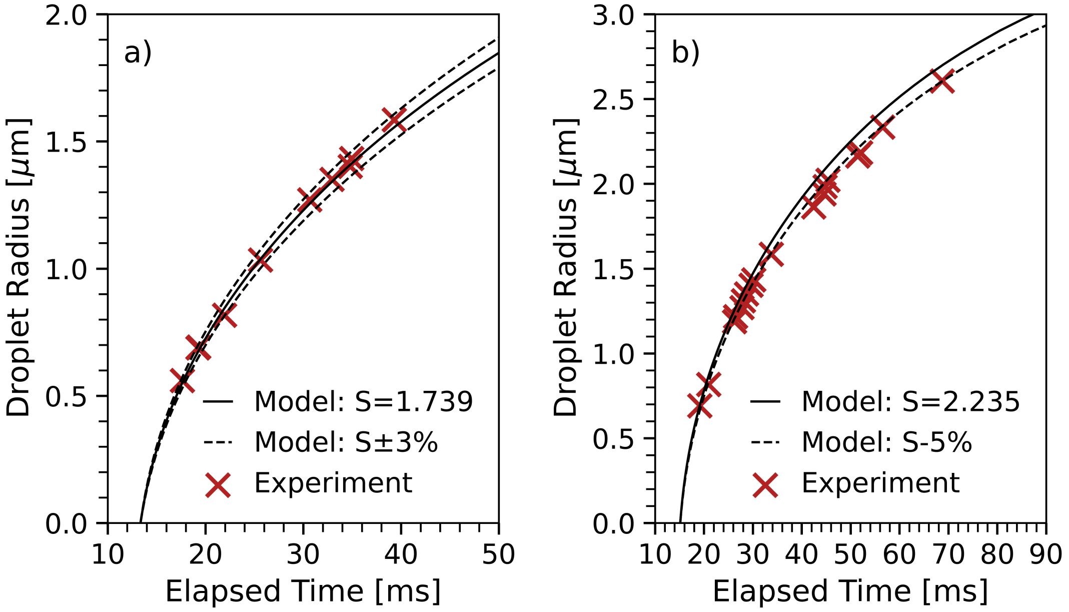

Figure S1: The figure compares the experimentally determined particle growth to theoretical predictions based on the model presented in Vesala et al.[2] The particle diameter is shown as function of the elapsed time after the expansion. The crosses represent experimental data. The solid lines indicate the theoretical particle growth at the measured saturation ratio S. The dashed lines represent theoretical calculations with an increased or reduced saturation ratio. Data shown in the two panels are related to the following experimental settings:

Panel a: $\mathrm{PET^P}$(-), $r_p = 3.6\ nm$, $T = 6.6°C$, $RH = 84.5\ \%$

Panel b: $\mathrm{PET^P}$(-), $r_p = 3.1\ nm$, $T = 6.1°C$, $RH = 93.0\ \%$

r_p is the radius of the seed particle, T depicts the temperature inside of the measurement chamber after the expansion and RH represents the saturation ratio of the aerosol exiting the humidifier.

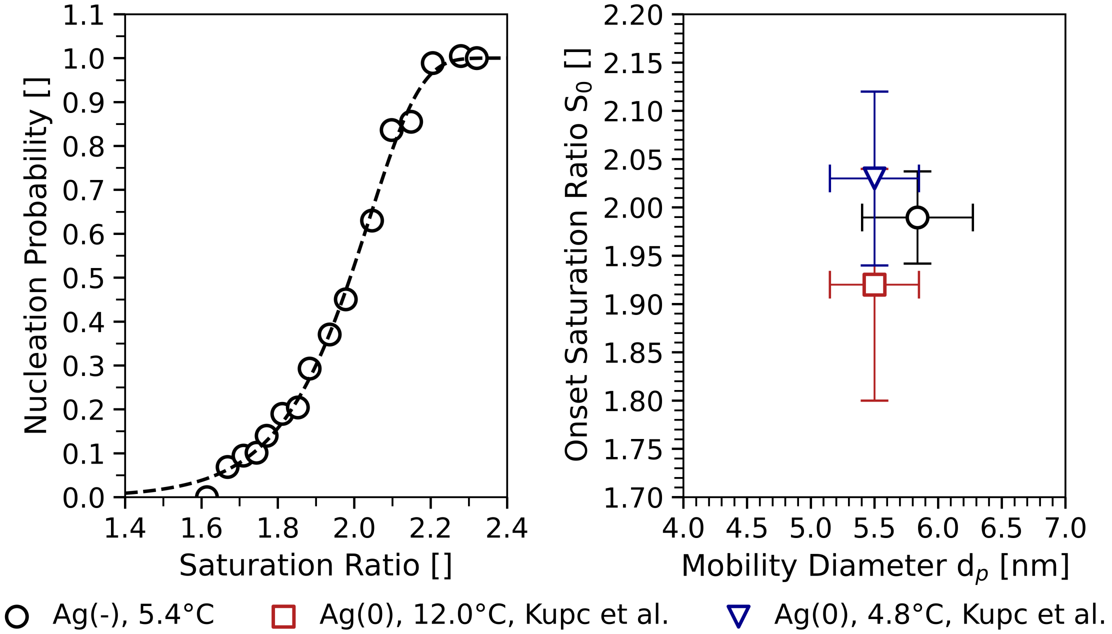

Figure S2: The figure summarizes the nucleation probabilities as well as the determined onset saturation ratio for Ag seeds. Panel a) shows the nucleation probabilities versus the determined saturation ratio of water vapor for negatively-charged Ag particles with a mobility diameter of 5.8 nm at a nucleation temperature of 5.4°C. Panel b) shows the comparison between the determined onset saturation ratio of the aforementioned seeds and data measured by Kupc et al.[3] using neutralized Ag seeds. The settings corresponding to the presented data can be found in Table S4, Table S5, and the Supplement to Kupc et al.[3]

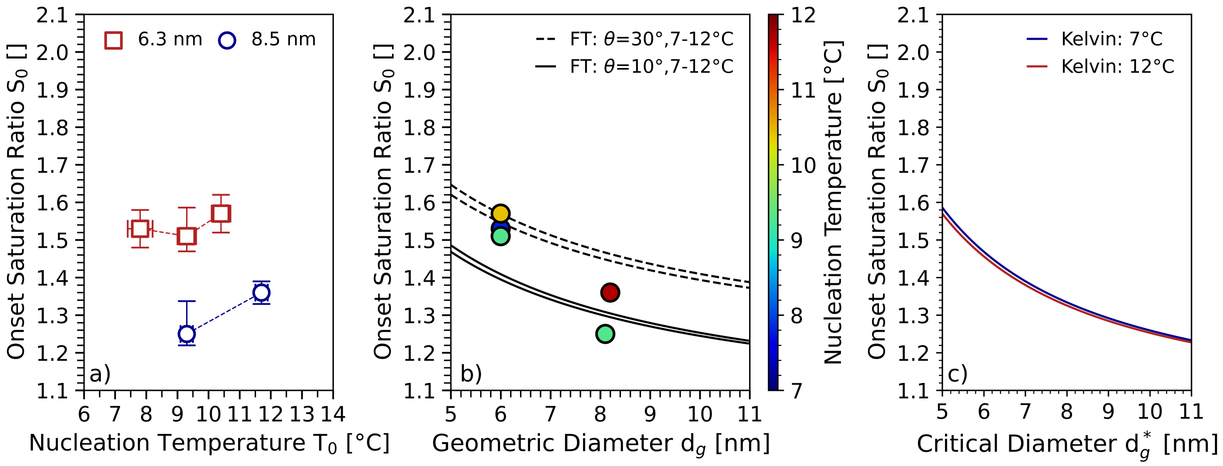

Figure S3: The figure shows the onset saturation ratios at different nucleation temperatures measured using PET^{C} seeds with diameters ranging from 6.3 to 8.5 nm. Panel a) presents the onset saturation ratio versus the nucleation temperature. The markers correspond to differently sized seed particles and the dashed line is solely a guide to the eye. Panel b) presents the onset saturation ratio as a function of the geometric diameter. The colors of the markers indicate the nucleation temperature. The solid and dashed lines correspond to theoretical onset saturation ratios calculated from Fletcher theory (FT) for different contact angles θ at a temperature range slightly exceeding the experimental nucleation temperatures. For the sake of readability, uncertainty bars were removed in panel b). Panel c) shows the onset saturation ratios as a function of the critical geometric diameter d_g^* as predicted by Kelvin equation.

C. Dataset

Table S4: The table summarizes the data presented in the figures. The onset saturation ratios S_0, the nucleation temperatures T_0, and the seed particle diameters d_p used in the experiments are shown. Uncertainties are marked with "Δ" and are presented with a corresponding sign. $\Delta S_{0,M}$ refers to the deviation of the experimental onset saturation ratio from the prediction based on the model presented by Vesala et al.[2] $\Delta S_{0,S}$ depicts the uncertainty arising from the RH sensor in use. Uncertainties without signs are symmetric.

Fig.	Seed	d_p $[nm]$	Δd_p $[nm]$	S_0	$\Delta S_{0,M}$ [%]	$\Delta S_{0,M}$	$\Delta S_{0,S}$ [%]	$\Delta S_{0,S}$	T_0 $[°C]$	ΔT_0 $[°C]$
2 a,b	PET^P(-)	10.1	0.7	1.30	+5	0.07	2.5	0.03	11.7	0.2
2 a,b	PET^P(-)	8.4	0.9	1.46	+5	0.07	2.4	0.04	11.2	0.3
2 a,b	PET^P(-)	7.2	0.6	1.64	-5	0.08	3.5	0.06	12.0	0.3
2 a,b	PET^P(-)	6.1	0.6	1.86	-5	0.09	3.4	0.06	10.0	0.3
3 a,b	PET^P(-)	6.1	0.6	1.78	3	0.05	2.4	0.04	7.3	0.3
3 a,b	PET^C(-)	6.3	0.7	1.53	3	0.05	2.5	0.04	7.8	0.4
3 a,b	Ag(-)	6.1	0.5	2.03	3	0.06	2.5	0.05	7.4	0.4
3 c	PET^P(-)	8.4	0.9	1.46	+5	0.07	2.4	0.04	11.2	0.3
3 c	PET^C(-)	8.5	0.9	1.36	-	-	2.4	0.03	11.7	0.2
4 a,b	PET^P(-)	7.2	0.6	1.48	5	0.07	2.6	0.04	6.8	0.2
4 a,b	PET^P(-)	7.2	0.6	1.52	3	0.05	2.4	0.04	9.0	0.3
4 a,b	PET^P(-)	7.2	0.6	1.64	-5	0.08	3.5	0.06	12.0	0.3
5 a,b	PET^P(-)	6.1	0.6	1.78	3	0.05	2.4	0.04	7.3	0.3
5 a,b	PET^P(-)	6.1	0.6	1.88	-5	0.09	3.4	0.06	9.3	0.3
5 a,b	PET^P(-)	6.1	0.6	1.86	-5	0.09	3.4	0.06	10.0	0.3
5 a,b	PET^P(-)	7.2	0.6	1.48	5	0.07	2.6	0.04	6.8	0.2
5 a,b	PET^P(-)	7.2	0.6	1.52	3	0.05	2.4	0.04	9.0	0.3
5 a,b	PET^P(-)	7.2	0.6	1.64	-5	0.08	3.5	0.06	11.9	0.3
5 a,b	PET^P(-)	8.4	0.7	1.34	+7	0.09	2.6	0.03	8.1	0.3
5 a,b	PET^P(-)	8.4	0.9	1.46	+5	0.07	2.4	0.04	11.2	0.3
5 a,b	PET^P(-)	8.4	0.9	1.56	-5	0.08	4.4	0.06	13.7	0.4
5 a,b	PET^P(-)	10.1	0.7	1.30	+5	0.07	2.5	0.03	11.7	0.2
5 a,b	PET^P(-)	10.1	0.7	1.36	-	-	3.5	0.05	15.3	0.2
6	PET^P(-)	7.2	0.6	1.48	5	0.07	2.6	0.04	6.8	0.2
6	PET^P(-)	7.2	0.6	1.52	3	0.05	2.4	0.04	9.0	0.3
6	PET^P(-)	7.2	0.6	1.64	-5	0.08	3.5	0.06	11.9	0.3
S2 a,b	Ag(-)	5.8	0.4	1.99	-	-	2.4	0.05	5.4	0.3
S3 a,b	PET^C(-)	6.3	0.7	1.53	3	0.05	2.5	0.04	7.8	0.4
S3 a,b	PET^C(-)	6.3	0.8	1.51	+5	0.08	2.4	0.04	9.3	0.3
S3 a,b	PET^C(-)	6.3	0.8	1.57	3	0.05	2.4	0.04	10.4	0.3
S3 a,b	PET^C(-)	8.4	0.8	1.25	+7	0.09	2.6	0.03	9.3	0.2
S3 a,b	PET^C(-)	8.5	0.9	1.36	-	-	2.4	0.03	11.7	0.2

Table S5: The table summarizes the experimental settings corresponding to the data presented in the figures. Q_f is the flow rate of compressed air passing the tube furnace, Q_{dil} depicts the flow rate of the dilution air fed into the system downstream of the tube furnace. The sample flow rate passing the DMA is therefore given by $Q_f + Q_{dil}$. Q_{dil2} refers to the (optional) dilution flow downstream of the DMA. Additionally, the table presents the DMA resolution R. Measurement uncertainties are presented in brackets.

Fig.	Seed	d_p $[nm]$	S_0	T_f $[°C]$	Q_f $[L \cdot min^{-1}]$	Q_{dil} $[L \cdot min^{-1}]$	Q_{dil2} $[L \cdot min^{-1}]$	R
2 a,b	PET^P(-)	10.1	1.30	200	0.50 (0.01)	1.30 (0.01)	2.30 (0.02)	15.3
2 a,b	PET^P(-)	8.4	1.46	210	0.80 (0.01)	2.40 (0.03)	-	9.3
2 a,b	PET^P(-)	7.2	1.64	224	1.20 (0.01)	1.50 (0.02)	0.50 (0.01)	11.8
2 a,b	PET^P(-)	6.1	1.86	204	0.70 (0.01)	2.20 (0.02)	0.70 (0.01)	10.6
3 a,b	PET^P(-)	6.1	1.78	220	1.50 (0.02)	1.70 (0.02)	1.30 (0.01)	9.6
3 a,b	PET^C(-)	6.3	1.53	182	2.00 (0.02)	1.20 (0.01)	-	9.3
3 a,b	Ag(-)	6.1	2.03	1000	1.50 (0.02)	0.80 (0.01)	-	13.3
3 c	PET^P(-)	8.4	1.46	210	0.80 (0.01)	2.40 (0.03)	-	9.3
3 c	PET^C(-)	8.5	1.36	200	1.60 (0.02)	1.60 (0.02)	1.00 (0.01)	9.3
4 a,b	PET^P(-)	7.2	1.48	228	1.20 (0.01)	1.50 (0.02)	0.50 (0.01)	12.4
4 a,b	PET^P(-)	7.2	1.52	228	1.20 (0.01)	1.50 (0.02)	0.50 (0.01)	11.4
4 a,b	PET^P(-)	7.2	1.64	224	1.20 (0.01)	1.50 (0.02)	0.50 (0.01)	11.8
5 a,b	PET^P(-)	6.1	1.78	220	1.50 (0.02)	1.70 (0.02)	1.30 (0.01)	9.6
5 a,b	PET^P(-)	6.1	1.88	204	0.70 (0.01)	2.20 (0.02)	0.70 (0.01)	10.2
5 a,b	PET^P(-)	6.1	1.86	204	0.70 (0.01)	2.20 (0.02)	0.70 (0.01)	10.6
5 a,b	PET^P(-)	7.2	1.48	228	1.20 (0.01)	1.50 (0.02)	0.50 (0.01)	12.4
5 a,b	PET^P(-)	7.2	1.52	228	1.20 (0.01)	1.50 (0.02)	0.50 (0.01)	11.4
5 a,b	PET^P(-)	7.2	1.64	224	1.20 (0.01)	1.50 (0.02)	0.50 (0.01)	11.8
5 a,b	PET^P(-)	8.4	1.34	200	0.70 (0.01)	2.70 (0.03)	1.90 (0.02)	12.8
5 a,b	PET^P(-)	8.4	1.46	210	0.80 (0.01)	2.40 (0.03)	-	9.3
5 a,b	PET^P(-)	8.4	1.56	210	0.80 (0.01)	2.30 (0.03)	-	9.6
5 a,b	PET^P(-)	10.1	1.30	200	0.50 (0.01)	1.30 (0.01)	2.30 (0.02)	15.3
5 a,b	PET^P(-)	10.1	1.36	200	0.50 (0.01)	1.30 (0.01)	2.30 (0.02)	15.3
6	PET^P(-)	7.2	1.48	228	1.20 (0.01)	1.50 (0.02)	0.50 (0.01)	12.4
6	PET^P(-)	7.2	1.52	228	1.20 (0.01)	1.50 (0.02)	0.50 (0.01)	11.4
6	PET^P(-)	7.2	1.64	224	1.20 (0.01)	1.50 (0.02)	0.50 (0.01)	11.8
S2 a,b	Ag(-)	5.8	1.99	1000	1.50 (0.02)	0.80 (0.01)	-	12.7
S3 a,b	PET^C(-)	6.3	1.53	182	2.00 (0.02)	1.20 (0.01)	-	9.3
S3 a,b	PET^C(-)	6.3	1.51	191	2.00 (0.02)	1.60 (0.02)	-	8.1
S3 a,b	PET^C(-)	6.3	1.57	191	2.00 (0.02)	1.60 (0.02)	-	8.1
S3 a,b	PET^C(-)	8.4	1.25	204	2.40 (0.02)	0.50 (0.01)	0.30 (0.003)	10.6
S3 a,b	PET^C(-)	8.5	1.36	200	1.60 (0.02)	1.60 (0.02)	1.00 (0.01)	9.3

D. Note on measurement uncertainties

The measurement uncertainties arising from the RH sensor[1] are crucial for the calculation of the overall uncertainties of the presented data. The manufacturer states a sensor accuracy of ± 1.8 %(RH) for a relative humidity ranging from 10 to 90 %. It is important to note, that the accuracy increases to ± 4 % for RH values > 90 % (s. Table S3). Uncertainties of the onset saturation ratios S_0 were determined using the sensor accuracy and calculations following the rules of Gaussian error propagation as well as by comparing the measured growth data to theoretical growth curves calculated using Vesala et al.[2] Initial saturation ratios of the aerosol downstream of the humidifier in the range of 0.8 to 0.9 have proven to yield the most accurate data (s. Figure S1). Lower RH values are connected to increasing deviations from the growth model; higher relative humidities are linked to higher sensor uncertainties.

E. Fletcher Theory (FT)

The curves in Figure 5, Figure 6 and Figure S3 corresponding to theoretical predictions from FT were calculated by following the approach outlined by Fletcher[4]:

1. The parameter x is given by the ratio of the seed particle diameter R to the critical cluster radius r^*,

$$x = \frac{R}{r^*}. \tag{1}$$

2. The parameter m is given by the cosine of the contact angle θ,

$$m = cos\theta. \tag{2}$$

3. The square of the logarithm of the saturation ratio S is given by,

$$(logS)^2 = \frac{8\pi \cdot V_l^2 \cdot \sigma^3 \cdot f(m,x)}{3k^3T^3 \cdot (60.1 + 4.606 \cdot log_{10}R)}, \tag{3}$$

where,

$$\begin{aligned} f(m,x) = \tfrac{1}{2} \cdot \Bigg\{ 1 + \left(\tfrac{1-mx}{g}\right)^3 + x^3 \cdot \left[2 - 3 \cdot \left(\tfrac{x-m}{g}\right) + \left(\tfrac{x-m}{g}\right)^3 \right] \\ + 3mx^2 \left(\tfrac{x-m}{g} - 1\right) \Bigg\}, \end{aligned} \tag{4}$$

with,

$$g = \sqrt{1 + x^2 - 2mx}. \tag{5}$$

Here, k depicts the Boltzmann constant, V_l is the volume of a water molecule in the bulk liquid, σ is the surface tension of the bulk liquid and T is the temperature.

4. Equation 4 is solved by successive approximation: Arbitrary values for x and m are inserted in Equation 4 and S is calculated.

5. Based on the resulting S, the seed particle diameter R can be calculated using,

$$R = x \cdot r^* = \frac{2x\sigma V_l}{kT \cdot logS}, \tag{6}$$

providing a better value for S when reinserted into Equation 3.

6. The process is repeated until it converges.

References

(1) Datasheet SHT7x (SHT71, SHT75) - Humidity and Temperature Sensor IC. 2010; https://sensirion.com/media/documents/9A2ADC83/61642805/Sensirion_Humidity_Sensors_SHT7x_Datasheet.pdf, accessed 2022-05-19.

(2) Vesala, T.; Kulmala, M.; Rudolf, R.; Vrtala, A.; Wagner, P. E. Models for condensational growth and evaporation of binary aerosol particles. *Journal of Aerosol Science* **1997**, *28 (4)*, 565–598, DOI: 10.1016/S0021-8502(96)00461-2.

(3) Kupc, A.; Winkler, P. M.; Vrtala, A.; Wagner, P. Unusual Temperature Dependence of Heterogeneous Nucleation of Water Vapor on Ag Particles. *Aerosol Science and Technology* **2013**, *47 (9)*, i–iv, DOI: 10.1080/02786826.2013.810330.

(4) Fletcher, N. H. Size Effect in Heterogeneous Nucleation. *The Journal of Chemical Physics* **1958**, *29 (3)*, 572–576, DOI: 10.1063/1.1744540.
